# Supplementary material for: The interactome of intact mitochondria by cross-linking mass spectrometry provides evidence for coexisting respiratory supercomplexes
Source: Mol Cell Proteomics. 2017 Dec 8;17(2):216–32. doi: 10.1074/mcp.RA117.000470 (PMC5795388; doi:10.1074/mcp.RA117.000470)
Supplement: Supplemental Data [file supp_RA117.000470_133922_0_supp_23978_fzffwf.zip › spectra_annotation/mito_DR_spectra_annotation/54-1-3-1-8-1.pdf]

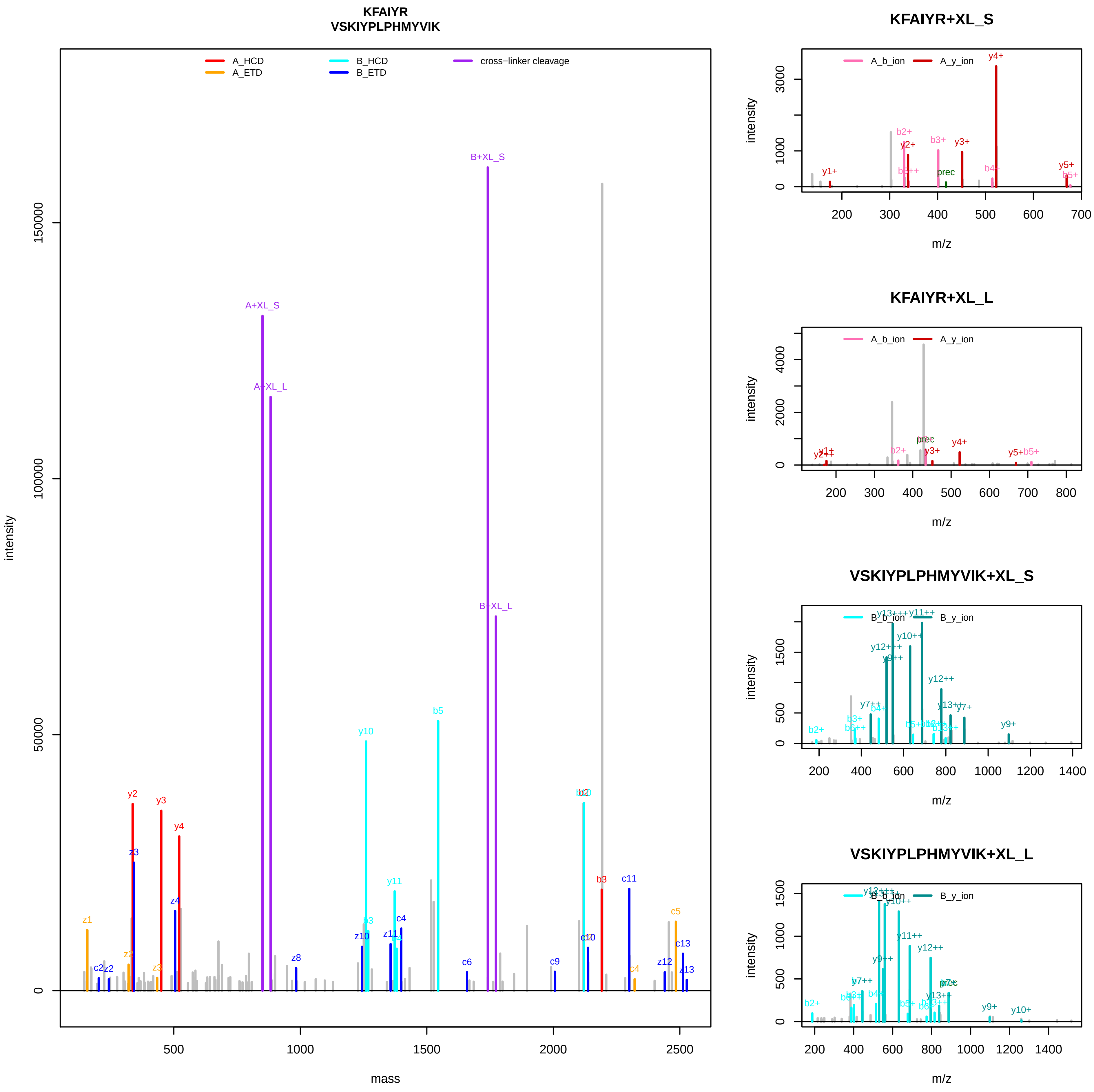
KFAIYR
VSKIYPLPHMYVIK
A_HCD
A_ETD
B_HCD
B_ETD
cross−linker cleavage
intensity
mass
0
50000
100000
150000
500
1000
1500
2000
2500
A+XL_S
A+XL_L
B+XL_S
B+XL_L
y2
y3
y4
z3
z4
z1
z2
c2
z8
y10
b5
y11
z10
z11
c4
c6
c9
c10
b3
c11
c5
c13
z12
z13
KFAIYR+XL_S
A_b_ion
A_y_ion
m/z
y1+
y2+
y3+
y4+
y5+
b2+
b3+
b4+
b5+
prec
KFAIYR+XL_L
VSKIYPLPHMYVIK+XL_S
B_b_ion
B_y_ion
y11++
y10++
y12++
y9++
y7++
y7+
y9+
b2+
b3+
b4+
VSKIYPLPHMYVIK+XL_L
y10+
